# Risk Factors of Posttraumatic Stress Disorder among Survivors after the 512 Wenchuan Earthquake in China

**DOI:** 10.1371/journal.pone.0022371

**Published:** 2011-07-25

**Authors:** Yuqing Zhang, Samuel M. Y. Ho

**Affiliations:** 1 Associate Professor, Key Laboratory of Behavioral Science, Institute of Psychology, Chinese Academy of Sciences, Beijing, China; 2 Professor, Department of Applied Social Studies, City University of Hong Kong, Hong Kong; Wayne State University, United States of America

## Abstract

This study investigated the psychological reactions of survivors of the 512 Wenchuan earthquake in China and the risk factors associated with those reactions. The Impact of Event Scale-Revised (IES-R), Type D Scale-14 (DS14), a self-developed trauma experience questionniare, and a demographic questionnaire were administered to 956 earthquake survivors (389 males and 567 females) in Mianzhu, one of the cities most affected by the earthquake. The results showed that postraumatic stress disorder (PTSD) symptoms affected 84.8% of survivors one to two months after the earthquake. Significant risk factors associated with PTSD symptoms included: (1) being female; (2) older age; (3) higher exposure to traumatic events during the earthquake; and (4) negative affect in Type-D personality.

## Introduction

Many studies of post-disaster psychological adjustment have been conducted during the past few decades. The majority suggest that the victims of disasters are likely to exhibit some degree of posttraumatic stress symptoms following the event [1], [2], [3], [4]. Moreover, it is proposed that if disaster victims do not receive proper posttraumatic psychological support within a certain period of time, their symptoms might deteriorate into more chronic psychological disorders, such as posttraumatic stress disorder (PTSD) [4]. Nevertheless, the large number of potential victims after a disaster makes it difficult or even impossible to provide psychological screening to all victims. Empirical studies that can help to identify victims with a higher risk of developing chronic psychological problems are needed. Kuo et al. [5] investigated the incidence of posttraumatic stress disorder among earthquake victims in Taiwan and reported that women tended to exhibit more PTSD symptoms than men. In addition, age and severity of injuries were both postively related to PTSD scores. Another study showed that posttraumatic symptoms were positively associated with the severity of the disaster, and the total number of family members lost as a result of the disaster [6].

Before 2008, there was a dearth of similar studies identifying the prevalence of posttraumatic stress disorders and the associated risk factors among people in China. One exception is a longitudinal study on earthquake-related PTSD in North China conducted by Wang XD and his colleagues [7]. The authors assessed victims of earthquake (n = 338) at 3 months and 9 months using the DSM-IV criteria, and reported a PTSD onset rate of 24.2% within a 9 months period. Interestingly, the authors also reported a trend of increasing PTSD onset rate over time. The authors explained their findings by hypothesizing that it might take time for the PTSD symptoms to develop. Also, they proposed that the prevalence of PTSD might be related to the post-disaster support provided. On May 12, 2008, a severe earthquake occurred in Wenchuan County of Sichuan Province in southwest China. This earthquake, often referred to as the 512 Wenchuan Earthquake, is the most devastating and destructive disaster in modern Chinese history. It was reported that 69,227 people died, 17,923 were missing, and 374,643 were injured in the disaster. Furthermore, 1,486,407 became homeless, and over 46.25 million people were affected by the earthquake. Many studies on the prevalence of psychological disorders (e.g. PTSD, depression and anxiety) and the risk factors associated with these disorders were conducted subsequent to this disaster [8]–[16]. Recently, Wang B and his colleagues [11] reported a study conducted among survivors of the 2008 Wenchuan earthquarke one month after the disaster and reported that 62.8% of their subjects met the criteria for PTSD diagnosis. Wang L and his colleagues [15], [16] used Impact of Event Scale to measure PTSD symptoms of the earthquake survivors at 2 months and 3 months post-earthquake and reported that 43% and 38% of their participants were suffering from probable PTSD respectively. Similarly, Kun et al. [13] reported a PTSD prevalence rate of 45.5% in a heavily damaged county of the earthquake area 6 months after the incident. After one year, a PTSD prevalence rate of 26.3% and 52.2% were reported in two independent studies [9], [12]. In addition, it was generally reported that female gender, older age, bodily injury and bereavement as well as earthquake exposure were important risks factors associated with PTSD among both adults [13], [15] and adolescents [8], [10].

One of the areas most affected by the Wenchuan earthquake was Mianzhu, a small city in Wenchuan County in which 11,119 people died, including 110 teachers and 1,100 students. On the second day after the earthquake, the government of Mianzhu City began to build temporary housing sites to provide basic accommodation for the city's homeless victims. The biggest site was based inside the sports center, in which over 10,000 homeless victims were assigned to live temporarily. The Institute of Psychology of the Chinese Academy of Science was one of the first organizations to send psychologists to the earthquake zone to provide psychological support to the earthquake survivors. On June 1, 2008, the Institute eastablished a work station in the sports center camp to provide psychological support and crisis intervention to the victims of Mianzhu and the surrounding areas. A study was conducted in June and July 2008, one to two months after the earthquake, to investigate the psychological effects of the earthquake on the survivors in Mianzhu city.

This paper reports some of the findings from the abovementioned study. In particular, it will focus on: (1) the prevalence of posttraumatic stress symptoms among the victims approximately one to months after the earthquake; and (2) how pre-disaster (e.g., gender) and during-disaster factors (e.g., exposure to traumatic incidents) affected posttraumatic stress symptoms.

## Methods

### Participants

A convenience sample of 956 service recipients of the Institute of Psychology, the Chinese Science Academy were recruited. All participants were survivors from Mianzhu, a city in Wenchuan County which was at the center of the earthquake area. At the time of this study (June and July 2008), all participants were living in temporary accommodation facilities provided by the Government of Mianzhu.

A total of 956 participants were recruited (men = 389, women = 567). Their ages ranged from 15 to 86 years (mean age = 36.69 years, S.D. = 15.79 years). Most of the participants were married (70.1%) and had middle school or above education level (60. 0%). Table 1 shows the demographic profile of the participants.

**Table 1:** Demographic profile of the participants (n = 956).

	Total	
	n	%
Gender		
Men	389	40.7
Women	567	59.3
Age		
Under 20 years	171	17.9
21–30 years	151	15.8
31–40 years	241	25.2
40–50 years	146	15.3
Above 50 years	188	19.7
Missing/Unknown	59	6.2
Marital Status		
Married	670	70.1
Single	278	29.1
Missing/Unknown	8	0.8
Education		
Elementary (Grade 1–6)	180	18.8
Middle School (Grade 7–9)	324	33.9
High School (Grade 10–12)	204	21.3
College or above	46	4.8
Missing/Unknown	202	21.1
Employment status		
Employed full-time in urban areas	182	19.0
Farmer in rural areas	261	27.3
Full-time students	165	17.3
Unemployed/retired	138	14.4
Missing/Unknown	210	21.9

### Procedures

Ethics approval was obtained from the Institutional Review Board of the Institute of Psychology, Chinese Academy of Sciences in Beijing, China. Only verbal consent was obtained from the participants because signing a consent form for research is not common in Mainland China and the study area. It would be very difficult to make the participants to understand that signing the forms has no other commitments and consequences besides the current research. We did not want to increase potential worries of the participants. In addition, the participants had just experienced a major disaster and it was necessary to complete the survey with minimal disturbances and as fast as possible during the acute phase of post-disaster management.

More than 100 voluntary psychological assistants working at the Mianzhu City Working Station in June and July, 2008 received training by the authors of this study on communication and interviewing skills before commencing data collection. The psychological assistants recruited participants during routine visits to the temporary housing tents of the participants to provide psychological support. Only one participant within a family was recruited for interview to ensure the independence of the data. The aims of the study were introduced and oral consent from the participants was obtained before data collection. Participants with secondary education or above were asked to read and complete the questionnaires by themselves. The psychological assistant was present during the whole data collection session to answer any queries from the participants. For those participants with elementary education or below, the questionaires were administered by the psychological assistant through face-to-face interview.

### Measures

#### Exposure to Trauma

A specially developed questionnaire was used to assess exposure to traumatic events during the disaster. Participants were asked whether they had (a) been trapped in the ruins of collapsed buildings/infrastructures; (b) suffered physical injures; (c) experienced bereavement; (d) witnessed the death of someone; (d) lost their home through it collapsing or being severely damaged; and (e) other traumatic experiences not mentioned above. Participants answered ‘yes’ or ‘no’ to each item. A total trauma exposure score was obtained by summing the responses of the six types of trauma exposure (yes = 1 and no = 0; range = 0–6), with higher scores implying more exposure to traumatic incidents during the earthquake. The Cronbach's alpha of this scale was .53 according to our sample.

#### Impact of Event Scale-Revised (IES-R)

The IES-R is probably the most widely used self-report measure in the field of traumatic stress [17], [18]. It was originally developed by Horowitz et al. [19] to monitor re-experiencing and avoidance tendencies after a traumatic event. The original IES consisted of 15 items with a 4-point Likert-style response format with two subscales: Intrusion and Avoidance. Later, another seven items on hyperarousal were added, based on the new diagnostic criteria of the DSM-IV [17], [20]. Thus, the IES-R consists of 22 items with a 5-point Likert-type scale ranging from 0 (not at all) to 4 (often). Three subscale scores can be obtained by summing the relevant item scores: Intrusion, Avoidance, and Hyperarousal. Furthermore, Creamer and his colleagues [18] proposed that individuals with an average IES-R score over 1.5 or a total IES-R score over 33 could be regarded as a “probable PTSD case”. These two cut-off points were used in the present study to identify PTSD cases. The reliability and validity of the Chinese version of the IES-R are reported to be good [21]. In this study, the Cronbach's alphas for the three subscales were 0.84 for Intrusion, 0.76 for Avoidance, and 0.81 for Arousal.

#### Type D Personality Scale -14 (DS-14)

The Type D personality scale measures the “distressed” personality [22], which is defined as the joint tendency towards negative affectivity (NA) (e.g., worry, irritability, and gloom) and social inhibition (SI) (e.g., reticence and a lack of self-assurance). High NA individuals usually experience more negative emotions, whereas high SI individuals tend to inhibit the expression of emotions or behavior and to avoid potential danger in social interactions [23]. The original scale for Type D Personality was a 16-item scale (DS-16) [24], which was later revised to 14 items (DS-14) by its original author [25]. The DS-14 is a brief, psychometrically validated measure of negative affectivity and social inhibition that can readily be incorporated in epidemiologic and clinical research. The reliability of the Chinese version of the DS-14 was 0.83 for the NA subscale and 0.72 for the SI subscale [26]. In the present study, Cronbach's alpha was 0.65 (NA) and 0.88 (SI).

#### Demographic data

This form was developed by the Institude of Psychology for the present study. Data collected included participants' age, gender, marrital status, and educational level.

### Statistical analysis

Descriptive statistics were provided first. Analyses of variance (ANOVAs), independent samples t-tests, and Pearson's correlation analyses were conducted to examine the relationships between demographic variables and measures, Finally, a hierarchical regression analysis was conducted with IES-R total score as the dependent variable. Demographic variables, total traumatic exposure score, and both NA and SI personality factors were entered as predictors. All analyses were carried out using Statistic Packages for Social Sciences (SPSS), Release Version 15.

## Results

### Prevalence of Traumatic Events During the Disaster and Post-disaster PTSD Symptoms

Regarding their earthquake experiences, the most common traumatic experiences encountered were losing one's home (i.e., house had collapsed or was severely damaged; 37.2%, n = 356) and being trapped under the ruins of collapsed buildings (34.7%, n = 332). Bereavement was the third most common trauma encountered, with 29.7% (n = 284) of our participants reporting that one or more of their family members had been killed by the earthquake. Prevalence rates of other traumatic events were: witnessing the death of someone (26.3%, n = 251); physical injuries (15.5% , n = 148) and other traumatic experiences (n = 321, 33.6%). No significant relationships were found between demographic factors and traumatic experiences, suggesting that everyone would have an equal chance of being affected by a major disaster such as the present earthquake.

Regarding the prevalence of PTSD, 790 participants (82.6%) had an IES-R total score higher than 33, and could be identified as “probable PTSD cases”. The average IES-R score in our sample was 1.97 (SD = .49), which was above the cutoff of 1.5 suggested by Creamer and his colleagues [18].

Next, we examined each of the demographic and psychological variables and their effect on post-earthquake PTSD symptoms. Due to the high percentage of missing values, marital status and employment status were not included in subsequent analyses.

### Gender

Table 2 shows the mean and SD of key variables by gender. Both men and women reported experiencing an average of about two traumatic events during the earthquake. However, both the total and subscale scores of the IES-R showed significant gender differences. The results indicate that compared with males, females tended to exhibit more intrusive thoughts and higher vigilance and avoidance behavior; consequently, females were at higher risk than males of developing PTSD after the earthquake. Subsequent analysis using the “probable PTSD case” cutoff score of 33 proposed by Creamer et al. [18] also showed that more women than men had an IES-R total score above the cutoff point in our sample (women: n = 495; 62.7%; men: n = 295; 37.3%; χ^2^(1) = 221..56, p<.001). Finally, our results also showed that women tended to have higher negative affectivity than men.

**Table 2:** Mean and standard deviation of IES-R scores and other psychological variables by gender.

Variables	Total	Male	Female	t value
	M (SD)	M (SD)	M (SD)	
Exposure to Trauma				
Total Trauma Exposure score	1.82 (1.49)	1.78 (1.47)	1.84 (1.50)	−.56
Impact of Event Scale – Revised (IES-R)				
Intrusion	16.64 (4.65)	15.42 (4.79)	17.47 (4.37)	−6.84**
Avoidance	14.98 (4.11)	14.10 (4.14)	15.58 (3.98)	−5.58**
Hyperarousal	11.77 (3.62)	10.66 (3.53)	12.52 (3.48)	−8.07**
Total IES-R score	43.39 (10.86)	40.19 (10.96)	45.58 (10.23)	−7.77**
Type D Personality Scale				
Social Inhibition	17.47 (3.36)	17.45 (3.23)	17.49 (3.44)	−.16
Negative Affect	16.99 (4.98)	16.54 (4.80)	17.30 (5.08)	−2.31*

*p<.05;

**p<.001.

### Age

Pearson product moment correlation analyses showed that age was positively and signficantly correlated with IES-R total score (r = .18, p<.001) and the three subscale scores: Intrusion (r = .21, p<.01), Avoidance (r = .09, p = .01), and Hyperarousal (r = .18, p<.01). Categorizing participants into different age groups revealed age group differences for all IES-R scores. Subsequent post hoc analyses suggested that participants in the 30–40 age group tended to report more PTSD symptoms, especially Intrusion (Table 3).

**Table 3:** Age group differences in IES-R.

	Age					
Variables	15–20	20–30	30–40	40–50	50+	F
Intrusion	13.58±4.33	16.42±4.01	18.07±4.44	17.53±4.25	17.11±4.75	29.27**
Avoidance	14.09±4.45	14.82±3.51	15.57±4.17	15.59±3.81	15.05±4.30	4.05**
Arousal	9.87±3.13	11.61±3.05	12.77±3.61	12.30±3.38	12.10±3.99	18.87**
IES Total	37.55±10.92	42.86±9.04	46.40±10.69	45.42±9.64	44.27±11.34	20.12**

### Type of Trauma Exposure

Our results showed that participants who reported being exposed to any one of the traumatic events included in this study tended to have higher IES-R total and subscale scores (Table 4). However, bereavement experience seemed to have less effect on PTSD symptoms, especially on intrusion. This result suggests that physical exposure to traumatic events might have a greater effect on subsequent reactions in the early stages of post-disaster adjustment.

**Table 4:** Relationship between PTSD symptoms and trauma exposure.

	IES Total			Intrusion			Avoidance			Arousal		
	Mean	(SD)	t-value	Mean	(SD)	t-value	Mean	(SD)	t-value	Mean	(SD)	t-value
Trapp in the Ruins												
Yes	46.17	(10.75)	5.89**	17.79	(4.64)	5.70**	15.82	(3.99)	4.62**	12.56	(3.65)	5.04**
No	41.90	(10.63)		16.02	(4.53)		14.54	(4.10)		11.34	(3.53)	
Physcial Injuries												
Yes	47.28	(11.98)	4.82**	18.16	(4.64)	4.45**	16.33	(4.72)	4.34**	12.79	(3.94)	3.82**
No	42.57	(10.63)		16.30	(4.66)		14.73	(3.98)		11.55	(3.55)	
Bereavement												
Yes	45.02	(10.82)	2.89**	16.93	(4.32)	1.12	15.89	(4.40)	4.41**	12.19	(3.62)	2.24*
No	42.78	(10.84)		16.56	(4.82)		14.61	(3.93)		11.62	(3.60)	
Wittnessed Death												
Yes	46.40	(11.23)	4.99**	18.06	(4.79)	5.49**	15.84	(4.24)	3.83**	12.50	(3.80)	3.56**
No	42.41	(10.58)		16.19	(4.53)		14.67	(4.05)		11.55	(3.52)	
Losing House												
Yes	45.62	(10.81)	4.95**	17.69	(4.59)	5.45**	15.46	(4.19)	2.75**	12.48	(3.77)	4.72**
No	42.06	(10.67)		16.01	(4.58)		14.70	(4.03)		11.34	(3.46)	
Other Traumatic Experiences												
Yes	44.79	(10.76)	2.75**	17.44	(4.70)	3.65**	15.17	(4.10)	1.02	12.18	(3.60)	2.41*
No	42.71	(10.93)		16.26	(4.62)		14.88	(4.13)		11.57	(3.63)	

Note:

**p<.01;

*p<.05.

### Degree of Trauma Exposure and Personality

Pearson product moment correlation coefficients are shown in Table 5. Total trauma exposure score was signficantly correlated with all of the IES-R scores, suggesting that the more traumatic events that participants were exposed to during the earthquake, the higher their risk of developing PTSD symptoms after the disaster.

**Table 5:** Correlations among the variables.

	Impact of Event Scale – Revised Score			
	IES-R Total	Intrusion	Avoidance	Hyperarousal
Total Trauma Exposoure Score	.30**	.29**	.24**	.25**
Social Inhibition	.18**	.16**	.13**	.19**
Negative Affect	.31**	.27**	.21**	.34**

**p<.001.

A significant positive correlation between IES-R total score and Negative Affect (NA) was found (r = .31, p<.001). NA also positively correlated with intrusion (r = .27, p<.001), avoidance (r = .21, p<.001), and arousal (r = .34, p<.001). These results suggest that people with NA personality were more likely to develop PTSD after the disaster. Social Inhibition (SI) was mildly but significantly correlated with IES-R (r = .18, p<.001) and also with other subscale scores.

### Regression Analyses

Table 6 presents the hierarchical regression results with IES-R total score as the dependent variable. Demographic variables, total traumatic exposure score, and both NA and SI personality factors were entered as predictors.

**Table 6:** Regression analysis with IES total score as dependent variable.

	B	SE B	β
*Step 1*			
Gender	6.25	.76	.28**
Age	.16	.02	.23**
F (2, 787) = 51.51, p<.001			
*Step 2*			
Gender	5.47	.70	.24**
Age	.15	.02	.21**
TTE	1.95	.23	.26**
NA	.57	.07	.25**
SI	−.02	.05	−.01
F (5, 784) = 55.73, p<.001			

Note: Gender coded as male = 0 and female = 1; TTE = Total Trauma Exposure score; NA = Negative Affect; SI = Social Inhibition.

**p<.001.

In step 1, the strongest predictors of the severity of posttraumatic stress reaction (IES-R score) were gender and age. Females had higher risk of developing PTSD symptoms. Also, participants aged 30 or above tended to report more PTSD symptoms than those below 30 years old. These two variables accounted for 11.6% of the total variance. In step 2, total trauma exposure score, negative affect, and social inhibition personality scores were entered. Inclusion of these three psychological variables significantly improved the predictive power of the regression equation (ΔR^2^ = .15, F Change = 51.88, p<.001). The significant individual predictors in the final equation were: gender, age, total trauma exposure score, and negative affect score.

## Discussion

Significant risk factors for post-earthquake PTSD symptoms identified in this study were: being female, of middle age (30–40 years), greater exposure to traumatic events during the disaster, and people with a negative affect personality. As discussed below, these factors are consistent with the existing literature on post-disaster adjustment. Our results provide further support for assessing post-disaster service needs and for identifying high-risk groups for preventative intervention in future disasters in China.

In the present study, the prevalence rate of posttraumatic stress symptoms one to two months after the earthquake was 82.6%, according to a cutoff score of 33 on the IES-R [18]. Previous studies have reported that the prevalence of PTSD in survivors of earthquakes ranges from 13% to 95%, depending on the severity of the earthquake, post-earthquake duration, and the measuring tools being used [6], [27]. As described earlier, other studies conducted among survivors of the Wenchuan earthquake reported a PTSD prevalence rate of 62.8% at one month [11], 43.0% at 2 months [15], and 37.8% at 3 months [16] post-earthquake. Our prevalence rate seems to be higher when compared to similar figures in these studies. However, the prevalence rate should be affected by the sample characteristics of a study, as well as the assessment methods being used. We adopted the criterion of Creamer [18] to determine probable PTSD cases which had not been used in previous studies before. Whether this criterion needs modification among Chinese populations should be investigated further in future studies. A detailed discussion of these issues, however, is beyond the scope of the present study.

Consistent with other studies [5], [8], [10], [13], [15], [28], [29], women tend to be more prone to developing PTSD symptoms than men. In this study, about 62.7% of women could be classified as “probable PTSD cases” compared to the corresponding figure of 37.3% for men. In addition, for all three domains of the IES-R – intrusion, avoidance, and hyperarousal – females had significantly higher scores than males.

As for the exposure indicators, the most common traumatic experiences were homes collapsing or being severely damaged (37.2%), being trapped under the ruins of collapsed buildings (34.7%), and bereavement (29.6%). Consistent with other studies, our results show that primary exposure to traumatic events during a disaster is an important risk factor for later development of PTSD symptoms. For example, Hsu et al. [30] found that the two risk factors for PTSD were being physically injured and experiencing the death of a close family member with whom they had lived. Besides these two traumatic experiences, our present study also identified seeing or touching a dead body as another important negative event related to later PTSD symptoms.

Regarding age, the results of this study showed that people in the 30–40 age group were the most affected by posttraumatic symptoms compared with other age groups. This result is consistent with the Green et al. [31] finding that the “middle-aged” adult group was more susceptible to posttraumatic symptoms after a natural disaster than were other ages. People in this middle age group are usually the main breadwinners of their families, especially men in a Chinese culture. They might consider that it is their responsibility to take care of their parents and children, and to provide them with a house, food, and security. The earthquake disrupted their ability to fulfill such responsibilities and led to more severe psychological problems. This may be due to various pressures, such as employment, family needs, and reconstruction of their homes [31], which might have led to a higher rate of PTSD symptoms among this group.

There are some limitations of this study. First, the post-disaster period of two months was short and it cannot be concluded that the predictors remain significantly related to PTSD for longer periods after the disaster. Second, we had to develop the trauma exposure items quickly and the six types of trauma included in this study are not comprehensive enough to cover all of the traumatic events encountered by the survivors during the earthquake. We have learned since this study that some people were stuck on the mountains during the earthquake. For instance, a 63 year-old man was stuck on a dangerous slope on a mountain for 456 hours before he was eventually rescued. Third, negative affect (NA) personality was found to be a significant factor related to PTSD symptoms. Since NA personality is a trait measure, we assumed that the NA personality style of a participant was not a result of the earthquake. Nevertheless, we could not excluded the possibility that the magnitude of the present earthquake was so huge that the NA personality of some participants might be the result of the disaster. Fourth, it should be noted that standardized post-disaster psychological care was not available in China before the 512 Wenchuan earthquake and only anecdotal interventions were conducted before and during the data collection period of this study. Psychological disaster intervention in the form of a complete continuum of care is developing in China and one of the authors (Zhang) is using the data and information from this study to help developing a standard of psychological care in China. The prevalence rate and relationships among variables may change due to enhancement of post-disaster psychological intervention in future. Finally, our study focused on earthquake survivors in China. The results cannot necessarily be generalized to other types of disaster or to earthquake survivors in other countries.

In conclusion, the prevalence of posttraumatic stress symptoms was high in Mianzhu city, an area that was suffering from serious damage one to two months after the Wenchuan earthquake in 2008. Risk factors for PTSD in survivors were female gender, marital status, more experience of traumatic events during the disaster, negative affect in Type D personality (NA), and elderly age.
